# 4-Ethyl-3-(3-pyrid­yl)-1*H*-1,2,4-triazole-5(4*H*)-thione 0.095-hydrate

**DOI:** 10.1107/S1600536808011744

**Published:** 2008-04-30

**Authors:** Muhammad Zareef, Muhammad Arfan, Rashid Iqbal, Masood Parvez

**Affiliations:** a Department of Chemistry, Quaid-i-Azam University, Islamabad 45320, Pakistan; b Department of Chemistry, The University of Calgary, 2500 University Drive NW, Calgary, Alberta, Canada T2N 1N4

## Abstract

The title compound, C_9_H_10_N_4_S·0.095H_2_O, consists of discrete 4-ethyl-3-(3-pyrid­yl)-1*H*-1,2,4-triazole-5(4H)-thione mol­ecules and a disordered mol­ecule of water of hydration with partial occupancy, lying on a twofold rotation axis. The dihedral angle between the pyridine and triazole rings is 41.73 (8)°. In the crystal structure, mol­ecules are hydrogen bonded *via* triazole NH groups and pyridyl N atoms, forming chains parallel to the *a* axis.

## Related literature

For related literature, see: Ahmad *et al.* (2001 ▶); Chai *et al.* (2003 ▶); Dege *et al.* (2004 ▶, 2005 ▶)); Demir *et al.* (2006 ▶); Dobosz *et al.* (2003 ▶); Hashimoto *et al.* (1990 ▶); Kanazawa *et al.* (1988 ▶); Mazur *et al.* (2006 ▶).
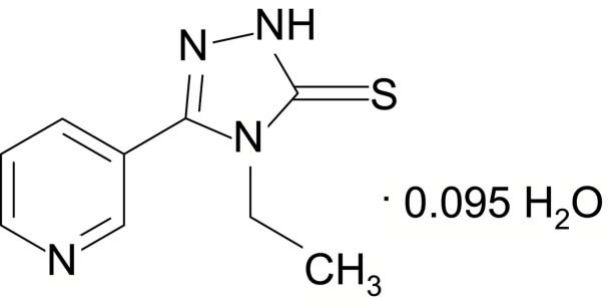


## Experimental

### 

#### Crystal data

C_9_H_10_N_4_S·0.095H_2_O
*M* *_r_* = 207.96
Monoclinic, 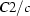

*a* = 14.076 (5) Å
*b* = 8.877 (5) Å
*c* = 16.216 (8) Å
β = 93.25 (3)°
*V* = 2023.0 (17) Å^3^
*Z* = 8
Mo *K*α radiation
μ = 0.29 mm^−1^
*T* = 173 (2) K
0.18 × 0.16 × 0.10 mm

#### Data collection

Nonius KappaCCD diffractometer
Absorption correction: multi-scan (*SORTAV*; Blessing, 1997 ▶) *T*_min_ = 0.950, *T*_max_ = 0.972
3392 measured reflections
2297 independent reflections
1616 reflections with *I* > 2σ(*I*)
*R*_int_ = 0.030

#### Refinement

*R*[*F* ^2^ > 2σ(*F* ^2^)] = 0.042
*wR*(*F* ^2^) = 0.107
*S* = 1.05
2297 reflections
137 parameters
H atoms treated by a mixture of independent and constrained refinement
Δρ_max_ = 0.24 e Å^−3^
Δρ_min_ = −0.25 e Å^−3^

### 

Data collection: *COLLECT* (Hooft, 1998 ▶); cell refinement: *DENZO* (Otwinowski & Minor, 1997 ▶); data reduction: *SCALEPACK* (Otwinowski & Minor, 1997 ▶); program(s) used to solve structure: *SHELXS97* (Sheldrick, 2008 ▶); program(s) used to refine structure: *SHELXL97* (Sheldrick, 2008 ▶); molecular graphics: *ORTEP-3 for Windows* (Farrugia, 1997 ▶); software used to prepare material for publication: *SHELXL97*.

## Supplementary Material

Crystal structure: contains datablocks global, I. DOI: 10.1107/S1600536808011744/lh2615sup1.cif

Structure factors: contains datablocks I. DOI: 10.1107/S1600536808011744/lh2615Isup2.hkl

Additional supplementary materials:  crystallographic information; 3D view; checkCIF report

## Figures and Tables

**Table 1:** Hydrogen-bond geometry (Å, °)

*D*—H⋯*A*	*D*—H	H⋯*A*	*D*⋯*A*	*D*—H⋯*A*
N2—H2⋯N4^i^	0.88 (2)	1.94 (2)	2.792 (3)	162 (2)

Symmetry code: (i) 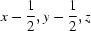.

**Fig. 1.**
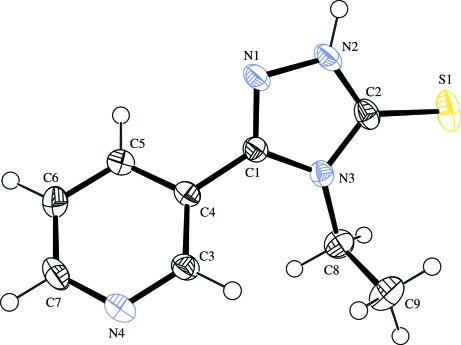
ORTEP-3 (Farrugia, 1997) drawing of (I) with displacement ellipsoids plotted at 50% probability level.

**Fig. 2.**
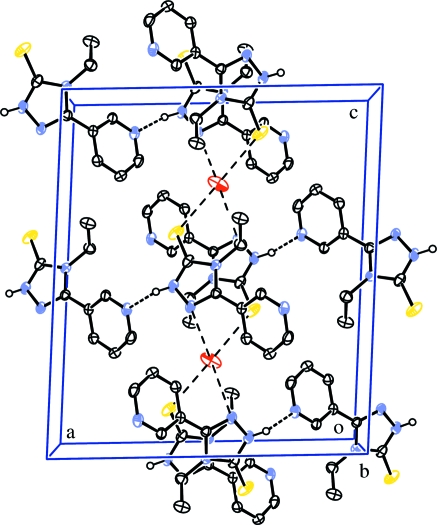
Unit cell packing of (I) showing hydrogen bonds with dashed lines; H-atoms not involved in H-bonds have been omitted.

## supplementary crystallographic information

### Comment

Disubstituted 1,2,4-triazoles and their derivatives are very important five membered heterocyclic compounds for their biological and pharmacological activities, such as antitumoral, inhibition of cholesterol, fungicidal, herbicidal, anticonvulsant (Kanazawa *et al.*, 1988; Chai *et al.*, 2003; Hashimoto *et al.*, 1990). Herein, we report the synthesis and crystal structure of the title compound, (I).

The structure of (I) is composed of independent molecules of 4-ethyl-2,4-dihydro-5-(3-pyridyl)-3*H*-1,2,4-triazole-3-thione (Fig. 1) and a disordered water of hydration with partial occupancy lying on a two-fold rotation axis. The bond distances and bond angles in (I) agree well with the corresponding bond distances and angles reported in some compounds closely related to (I) (*e.g.*, Dege *et al.*, 2004, 2005; Mazur *et al.*, 2006; Dobosz *et al.*, 2003; Demir, *et al.*, 2006). The mean-planes formed by the pyridyl and triazole rings in (I) lie at 41.73 (8)° with respect to each other while the mean-plane of the ethyl group is inclined with the triazole ring at 80.39 (13)°.

The water of hydration is surrounded by four molecules of (I) with S1···O1 and N1···O1 separation of 3.074 (4) and 3.382 (9) Å, respectively (Fig. 2). The molecules of (I) are hydrogen bonded *via* N2—H2···N4 forming chains lying parallel to the *a* axis (details of hydrogen bonding geometry have been given in Table 1). The shortest distance between the centroids of pyridyl and triazole rings from two different molecules lying about inversion centers is 4.350 (3) Å.

### Experimental

The title compound was prepared from the corresponding thiosemicarbazide by following the reported procedure (Ahmad *et al.*, 2001). 4-Ethyl-1-(2-pyridoyl)thiosemicarbazide (12 mmol) was dissolved in an aqueous 4 N sodium hydroxide solution (65 ml). The solution was heated to reflux for 11.5 h, cooled and filtered. The filtrate was acidified to pH of 4–5, with 4 N hydrochloric acid. The solid product was filtered off, washed with water and recrystallized from aqueous ethanol (60%). Crystals of (I) were grown by slow evaporation of the ethanol over 15 days at room temperature (yield 72%).

### Refinement

Though all the H atoms could be distinguished in the difference Fourier map the H-atoms bonded to C-atoms were included at geometrically idealized positions and refined in riding-model approximation with the following constraints: pyridyl, methyl and methylene C—H distances were set to 0.95, 0.98 and 0.99 Å, respectively; in all these instances *U*_iso_(H) = 1.2 *U*_eq_(C). H-atom bonded to N2 was taken from the difference map and was allowed to refine with *U*_iso_ = 1.2 times *U*_eq_ of N2. Towards the end of the refinement, a difference Fourier map revealed a peak that was included in the refinement as an O-atom the site occupancy factor of which refined to 0.095; its s.o.f. was fixed at that value during the final rounds of calculations. The H-atoms bonded to the O atom of the water molecule could not be located and were not included in the refinement but are included in the molecular formula. The atmospheric moisture was assumed to be the source of this partially occupied water of hydration. The final difference map was free of any chemically significant features.

### Crystal data

**Table:**

C_9_H_10_N_4_S·0.095H_2_O	*F*_000_ = 872
*M**_r_* = 207.96	*D*_x_ = 1.366 Mg m^−^^3^
Monoclinic, *C*2/*c*	Melting point = 440–441 K
Hall symbol: -C 2yc	Mo *K*α radiation λ = 0.71073 Å
*a* = 14.076 (5) Å	Cell parameters from 3392 reflections
*b* = 8.877 (5) Å	θ = 3.6–27.4º
*c* = 16.216 (8) Å	µ = 0.29 mm^−^^1^
β = 93.25 (3)º	*T* = 173 (2) K
*V* = 2023.0 (17) Å^3^	Block, colorless
*Z* = 8	0.18 × 0.16 × 0.10 mm

### Data collection

**Table:**

Nonius KappaCCD diffractometer	2297 independent reflections
Radiation source: fine-focus sealed tube	1616 reflections with *I* > 2σ(*I*)
Monochromator: graphite	*R*_int_ = 0.030
*T* = 173(2) K	θ_max_ = 27.4º
ω and φ scans	θ_min_ = 3.6º
Absorption correction: Multi-scan(SORTAV; Blessing, 1997)	*h* = −17→18
*T*_min_ = 0.950, *T*_max_ = 0.972	*k* = −11→9
3392 measured reflections	*l* = −20→20

### Refinement

**Table:**

Refinement on *F*^2^	Hydrogen site location: inferred from neighbouring sites
Least-squares matrix: full	H atoms treated by a mixture of independent and constrained refinement
*R*[*F*^2^ > 2σ(*F*^2^)] = 0.042	*w* = 1/[σ^2^(*F*_o_^2^) + (0.0405*P*)^2^ + 1.636*P*] where *P* = (*F*_o_^2^ + 2*F*_c_^2^)/3
*wR*(*F*^2^) = 0.107	(Δ/σ)_max_ < 0.001
*S* = 1.05	Δρ_max_ = 0.24 e Å^−^^3^
2297 reflections	Δρ_min_ = −0.25 e Å^−^^3^
137 parameters	Extinction correction: SHELXL97 (Sheldrick, 2008), Fc^*^=kFc[1+0.001xFc^2^λ^3^/sin(2θ)]^-1/4^
Primary atom site location: structure-invariant direct methods	Extinction coefficient: 0.0038 (9)
Secondary atom site location: difference Fourier map	

### Special details

**Table:**

Geometry. All e.s.d.'s (except the e.s.d. in the dihedral angle between two l.s. planes) are estimated using the full covariance matrix. The cell e.s.d.'s are taken into account individually in the estimation of e.s.d.'s in distances, angles and torsion angles; correlations between e.s.d.'s in cell parameters are only used when they are defined by crystal symmetry. An approximate (isotropic) treatment of cell e.s.d.'s is used for estimating e.s.d.'s involving l.s. planes.
Refinement. Refinement of *F*^2^ against ALL reflections. The weighted *R*-factor *wR* and goodness of fit *S* are based on *F*^2^, conventional *R*-factors *R* are based on *F*, with *F* set to zero for negative *F*^2^. The threshold expression of *F*^2^ > σ(*F*^2^) is used only for calculating *R*-factors(gt) *etc*. and is not relevant to the choice of reflections for refinement. *R*-factors based on *F*^2^ are statistically about twice as large as those based on *F*, and *R*- factors based on ALL data will be even larger.

### Fractional atomic coordinates and isotropic or equivalent isotropic displacement parameters (Å^2^)

**Table:**

	*x*	*y*	*z*	*U*_iso_*/*U*_eq_	Occ. (<1)
S1	−0.12974 (4)	0.05644 (7)	−0.11164 (3)	0.0415 (2)	
O1	0.0000	−0.0319 (16)	−0.2500	0.070 (4)	0.19
N1	−0.07296 (10)	0.26787 (18)	0.09536 (9)	0.0256 (4)	
N2	−0.13013 (11)	0.19176 (19)	0.03795 (10)	0.0276 (4)	
H2	−0.1854 (16)	0.158 (3)	0.0530 (12)	0.033*	
N3	0.00251 (10)	0.22103 (18)	−0.01818 (9)	0.0228 (4)	
N4	0.21945 (11)	0.53359 (19)	0.10227 (10)	0.0290 (4)	
C1	0.00713 (12)	0.2838 (2)	0.05951 (10)	0.0217 (4)	
C2	−0.08639 (13)	0.1576 (2)	−0.03071 (12)	0.0267 (4)	
C3	0.14695 (12)	0.4604 (2)	0.06268 (11)	0.0246 (4)	
H3	0.1343	0.4794	0.0055	0.029*	
C4	0.08963 (12)	0.3582 (2)	0.10142 (11)	0.0220 (4)	
C5	0.10899 (13)	0.3310 (2)	0.18541 (11)	0.0263 (4)	
H5	0.0714	0.2616	0.2140	0.032*	
C6	0.18343 (14)	0.4061 (2)	0.22654 (12)	0.0313 (5)	
H6	0.1977	0.3897	0.2838	0.038*	
C7	0.23642 (13)	0.5050 (2)	0.18282 (12)	0.0313 (5)	
H7	0.2878	0.5559	0.2113	0.038*	
C8	0.07912 (14)	0.1998 (2)	−0.07478 (12)	0.0300 (5)	
H8A	0.0756	0.0962	−0.0973	0.036*	
H8B	0.1412	0.2110	−0.0436	0.036*	
C9	0.07367 (17)	0.3113 (3)	−0.14570 (13)	0.0406 (6)	
H9A	0.1216	0.2857	−0.1849	0.049*	
H9B	0.0856	0.4132	−0.1243	0.049*	
H9C	0.0102	0.3072	−0.1738	0.049*	

### Atomic displacement parameters (Å^2^)

**Table:**

	*U*^11^	*U*^22^	*U*^33^	*U*^12^	*U*^13^	*U*^23^
S1	0.0476 (4)	0.0346 (4)	0.0398 (3)	−0.0034 (3)	−0.0202 (3)	−0.0060 (2)
O1	0.107 (12)	0.043 (9)	0.063 (9)	0.000	0.034 (9)	0.000
N1	0.0194 (8)	0.0271 (10)	0.0300 (8)	−0.0060 (7)	−0.0022 (6)	0.0005 (7)
N2	0.0201 (8)	0.0283 (10)	0.0339 (9)	−0.0080 (7)	−0.0032 (7)	0.0017 (7)
N3	0.0228 (8)	0.0221 (9)	0.0232 (8)	−0.0008 (7)	−0.0026 (6)	0.0016 (6)
N4	0.0220 (8)	0.0282 (10)	0.0367 (9)	−0.0042 (7)	0.0002 (7)	0.0000 (7)
C1	0.0209 (9)	0.0215 (10)	0.0225 (9)	−0.0007 (8)	−0.0008 (7)	0.0031 (7)
C2	0.0256 (10)	0.0221 (11)	0.0315 (10)	−0.0015 (8)	−0.0076 (8)	0.0044 (8)
C3	0.0199 (9)	0.0269 (11)	0.0267 (9)	−0.0012 (8)	−0.0001 (7)	0.0024 (8)
C4	0.0180 (9)	0.0212 (10)	0.0267 (9)	−0.0001 (7)	0.0005 (7)	−0.0007 (8)
C5	0.0234 (10)	0.0288 (11)	0.0268 (9)	−0.0024 (8)	0.0027 (7)	−0.0005 (8)
C6	0.0298 (10)	0.0399 (13)	0.0237 (9)	−0.0020 (9)	−0.0034 (8)	−0.0027 (9)
C7	0.0226 (10)	0.0333 (12)	0.0373 (11)	−0.0047 (9)	−0.0052 (8)	−0.0081 (9)
C8	0.0316 (10)	0.0289 (12)	0.0299 (10)	0.0029 (9)	0.0044 (8)	−0.0006 (9)
C9	0.0540 (14)	0.0397 (14)	0.0289 (11)	0.0010 (11)	0.0096 (10)	0.0024 (10)

### Geometric parameters (Å, °)

**Table:**

S1—C2	1.676 (2)	C3—C4	1.388 (3)
S1—O1	3.074 (4)	C3—H3	0.9500
O1—S1^i^	3.074 (4)	C4—C5	1.395 (3)
O1—N1^ii^	3.382 (9)	C5—C6	1.381 (3)
N1—C1	1.305 (2)	C5—H5	0.9500
N1—N2	1.373 (2)	C6—C7	1.375 (3)
N2—C2	1.337 (3)	C6—H6	0.9500
N2—H2	0.88 (2)	C7—H7	0.9500
N3—C1	1.376 (2)	C8—C9	1.516 (3)
N3—C2	1.377 (2)	C8—H8A	0.9900
N3—C8	1.467 (2)	C8—H8B	0.9900
N4—C7	1.339 (3)	C9—H9A	0.9800
N4—C3	1.342 (2)	C9—H9B	0.9800
C1—C4	1.469 (2)	C9—H9C	0.9800
			
C2—S1—O1	120.21 (15)	C5—C4—C1	118.66 (16)
S1—O1—S1^i^	150.4 (5)	C6—C5—C4	119.26 (18)
S1—O1—N1^ii^	77.57 (10)	C6—C5—H5	120.4
S1^i^—O1—N1^ii^	122.08 (18)	C4—C5—H5	120.4
C1—N1—N2	103.82 (16)	C7—C6—C5	118.57 (18)
C2—N2—N1	113.35 (16)	C7—C6—H6	120.7
C2—N2—H2	127.4 (14)	C5—C6—H6	120.7
N1—N2—H2	118.2 (14)	N4—C7—C6	123.51 (17)
C1—N3—C2	107.22 (15)	N4—C7—H7	118.2
C1—N3—C8	128.79 (15)	C6—C7—H7	118.2
C2—N3—C8	123.33 (16)	N3—C8—C9	112.54 (17)
C7—N4—C3	117.62 (17)	N3—C8—H8A	109.1
N1—C1—N3	111.53 (16)	C9—C8—H8A	109.1
N1—C1—C4	121.52 (16)	N3—C8—H8B	109.1
N3—C1—C4	126.94 (16)	C9—C8—H8B	109.1
N2—C2—N3	104.00 (16)	H8A—C8—H8B	107.8
N2—C2—S1	127.52 (15)	C8—C9—H9A	109.5
N3—C2—S1	128.46 (16)	C8—C9—H9B	109.5
N4—C3—C4	123.08 (17)	H9A—C9—H9B	109.5
N4—C3—H3	118.5	C8—C9—H9C	109.5
C4—C3—H3	118.5	H9A—C9—H9C	109.5
C3—C4—C5	117.95 (16)	H9B—C9—H9C	109.5
C3—C4—C1	123.31 (16)		
			
C2—S1—O1—S1^i^	−60.83 (15)	O1—S1—C2—N3	5.5 (3)
C2—S1—O1—N1^ii^	74.7 (3)	C7—N4—C3—C4	0.2 (3)
C1—N1—N2—C2	1.6 (2)	N4—C3—C4—C5	−0.1 (3)
N2—N1—C1—N3	0.1 (2)	N4—C3—C4—C1	176.65 (18)
N2—N1—C1—C4	−178.76 (16)	N1—C1—C4—C3	−137.4 (2)
C2—N3—C1—N1	−1.8 (2)	N3—C1—C4—C3	43.9 (3)
C8—N3—C1—N1	−172.57 (17)	N1—C1—C4—C5	39.3 (3)
C2—N3—C1—C4	177.06 (18)	N3—C1—C4—C5	−139.4 (2)
C8—N3—C1—C4	6.3 (3)	C3—C4—C5—C6	0.2 (3)
N1—N2—C2—N3	−2.7 (2)	C1—C4—C5—C6	−176.73 (18)
N1—N2—C2—S1	175.75 (14)	C4—C5—C6—C7	−0.3 (3)
C1—N3—C2—N2	2.6 (2)	C3—N4—C7—C6	−0.4 (3)
C8—N3—C2—N2	174.01 (16)	C5—C6—C7—N4	0.4 (3)
C1—N3—C2—S1	−175.82 (15)	C1—N3—C8—C9	−105.1 (2)
C8—N3—C2—S1	−4.4 (3)	C2—N3—C8—C9	85.4 (2)
O1—S1—C2—N2	−172.6 (3)		

Symmetry codes: (i) −*x*, *y*, −*z*−1/2; (ii) −*x*, −*y*, −*z*.

### Hydrogen-bond geometry (Å, °)

**Table:**

*D*—H···*A*	*D*—H	H···*A*	*D*···*A*	*D*—H···*A*
N2—H2···N4^iii^	0.88 (2)	1.94 (2)	2.792 (3)	162 (2)

Symmetry codes: (iii) *x*−1/2, *y*−1/2, *z*.

## References

[bb1] Ahmad, R., Iqbal, R., Akhtar, R. H., Haq, Z. U., Duddeck, H., Stefaniak, L. & Sitkowski, J. (2001). *Nucleosides Nucleotides Nucleic Acids*, **20**, 1671–1682. 10.1081/NCN-100105903 11580193

[bb2] Blessing, R. H. (1997). *J. Appl. Cryst.* **30**, 421–426.

[bb3] Chai, B., Qian, X., Cao, S., Liu, H. & Song, G. (2003). *Arkovic.* **ii**, 141–145.

[bb4] Dege, N., Cetin, A., Cansiz, A., Sekerci, M., Kazaz, C., Dincer, M. & Buyukgungor, O. (2004). *Acta Cryts.* **E60**, o1883–o1885.

[bb5] Dege, N., Özdemir, N., Çetin, A., Cansız, A., Şekerci, M. & Dinçer, M. (2005). *Acta Cryst.* E**61**, o17–o19.

[bb6] Demir, S., Dinçer, M., Çetin, A., Dayan, O. & Cansız, A. (2006). *Acta Cryst.* E**62**, o2198–o2199.

[bb7] Dobosz, M., Pitucha, M., Dybala, I. & Koziol, A. E. (2003). *Collect. Czech. Chem. Commun.* **68**, 792–800.

[bb8] Farrugia, L. J. (1997). *J. Appl. Cryst.* **30**, 565.

[bb9] Hashimoto, F., Sugimoto, C. & Hayashi, H. (1990). *Chem. Pharm. Bull.* **38**, 2532–2536. 10.1248/cpb.38.2532 2285988

[bb10] Hooft, R. (1998). *COLLECT* Nonius BV, Delft, The Netherlands.

[bb11] Kanazawa, S., Driscoll, M. & Struhl, K. (1988). *Mol. Cell. Biol.* **8**, 644–673. 10.1128/mcb.8.2.664-673.1988 PMC363192 3280970

[bb12] Mazur, L., Koziol, A. E., Maliszewska-Guz, A., Wujec, M., Pitucha, M. & Dobosz, M. (2006). *Z. Kristallogr. New Cryst. Struct.* **221**, 151–152.

[bb13] Otwinowski, Z. & Minor, W. (1997). *Methods in Enzymology*, Vol. 276, *Macromolecular Crystallography*, Part A, edited by C. W. Carter Jr. and R. M. Sweet, pp. 307–326. New York: Academic Press.

[bb14] Sheldrick, G. M. (2008). *Acta Cryst.* A**64**, 112–122. 10.1107/S0108767307043930 18156677

